# Auxiliary Hospitals and Their Uses

**Published:** 1917-09-22

**Authors:** 


### AUXILIARY HOSPITALS AND THEIR USES.

A correspondent in the *Times* has suggested that a large class of soldier patients are too well to remain in the big military hospitals, but not well enough to be passed on to duty or Command depôts: they are sent on to the auxiliary hospitals, which are small affairs where the men get almost too much kindness and comfort, and certainly too little of the skilled physiotherapy which the majority need. These hospitals cannot have provision, still less the necessary experience, for this special form of treatment. "The result," says the correspondent, "is waste of doctor power, waste of nursing power, waste of money in effort and upkeep, and waste of military man-power through delay in the recovery of patients." He suggests further that these hundreds of small hospitals now scattered broadcast over the country should be entirely replaced by a few large central hospitals for convalescents, to supply the link between the general hospital on the one hand and the Command depôt or full duty on the other. These would be able to give the nursing and comfort that would still be required, to employ specialists in an economical manner, to pay adequate attention to specialised treatment, and would do in every way more than the present auxiliary hospitals with much smaller staff and for much smaller expense.

This is all very well, but the correspondent seems to leave out of sight that the provision of very large buildings specially constructed or specially altered in construction for the purposes of hospitals would incur an expense still greater than that which is now incurred in the administration of smaller auxiliary hospitals. We do not agree that the men who have been wounded in this war and have offered their lives and their health to the country can be treated with too much kindness, or can receive too much comfort. It may be that they get too little of the skilled physiotherapy that some of them need, and it is certainly true that some auxiliary hospitals are administered extravagantly; but it is not the auxiliary hospitals alone that are thus administered. Much has been done to enforce economy in the administration of the larger hospitals, but much still remains to be done.

It would seem to be a better plan to collect the auxiliary hospitals into single districts—to group them round a large general hospital in an important town, so that the specialists on the staff of the hospital could be employed or could give their services to the auxiliary hospitals also in the special matters necessary for completing the cure of convalescents.

The general rule is that the large institutions, whatever their kind—whether hospitals, asylums, schools or barracks—are apt to treat their inmates too much in the gross and to give them too little individual attention.

